# Metabolic rate does not explain performance on a short-term memory task or personality traits in juvenile chickens (*Gallus gallus domesticus*)

**DOI:** 10.1098/rsos.221650

**Published:** 2023-09-13

**Authors:** Cecylia M. Watrobska, Grégoire Pasquier, Ellouise Leadbeater, Steven J. Portugal

**Affiliations:** Department of Biological Sciences, School of Life and Environmental Sciences, Royal Holloway University of London, Egham TW20 0EX, UK

**Keywords:** cognition, dominance, *Gallus*, radial arm maze, respirometry

## Abstract

Metabolic rate determines life processes and the physiological requirements of an individual, and has recently been implicated as a driver of inter-individual variation in behaviour, with positive correlations associated with boldness, exploration and aggressive behaviours being recorded. While the link between metabolism and personality has been explored, little is known about the influence of metabolism on cognitive abilities. Here we used juvenile female chickens (*Gallus gallus domesticus*) to investigate the relationships between metabolic rate at rest, short-term memory, personality, and dominance. Resting metabolic rates of the chicks were measured over a three-week period, concurrently with measures of short-term memory using an analogue of the radial arm maze. We also measured latency to leave the shelter (boldness), neophobia (fear of novel objects) and dominance within a group, both before and after short-term memory trials. We found that metabolic rate did not explain inter-individual differences in short-term memory, personality traits or dominance, suggesting that energy allocated to these traits is independent of individual metabolic rate, and providing evidence for the independent energy-management hypothesis. Differences in short-term memory were also not explained by boldness or neophobia. Variation in behaviour in chicks, therefore, appears to be driven by separate, currently unknown variables.

## 1. Introduction

Energy is the currency of life, and an organism's metabolism acquires, transforms and allocates energy to drive all processes in the body [1]. Variation in metabolic rate-evident at both the inter- and intra- specific level [2,3]–drives differences in aspects of life-history traits such as growth rate [4,5], reproductive investment [6], and behaviour [3]. The relationship between behaviour and metabolic rate is complex, as all behaviours are energetically costly; some provide an instantaneous net gain (e.g. foraging), while others are associated with a net loss (e.g. defence of a territory) of energy to the organism [3].

An area that has received comparatively little attention is the interaction between metabolic rate and animal personality. Personality defines a set of correlated behaviours consistently displayed by an individual in different contexts and over time [7]. Aspects of personality include the scale of slow to fast exploration [8], where bolder individuals show faster tendencies to explore a novel or unfamiliar environment. Greater boldness generally correlates positively with aggression [7], activity levels [9] and dominance within a group [10]. Drivers of differences in personality between individuals are most likely multi-factorial, and include genetic differences [8,11], prior experiences [12] and the environment [8]. Metabolic rate has also been associated with differences in personality [13]. For example, bolder individuals of common carp (*Cyprinus caprio*) that were more willing to take risks had a higher standard metabolic rate [14], as did bolder individuals of Atlantic salmon (*Salmo salar*) that spent more time exploring uncovered areas of their tanks [15]. Similarly, more dominant male white-throated dippers (*Cinclus cinclus*) had a higher basal metabolic rate [16]. However, other studies have found no relationship between metabolic rate and boldness [17] or dominance [18,19], suggesting the relationship is complex and may be context-dependent.

The association between cognitive elements, personality, metabolism and dominance rank is typically complex. Several energetic-based models predict the potential relationships between metabolic rate and personality traits, and in turn dominance, under various scenarios, and these probably apply to cognition also [13,20]. The *allocation model* suggests variation in metabolic rate is not associated with changes in behaviours relating to energy gain; individuals with higher metabolic costs are less likely to invest in foraging behaviours, gain dominance, and exhibit overall boldness personality traits, owing to their energetically costly nature [13]. By contrast, a low metabolic rate may permit individuals to engage in costly behaviours, such as aggression, because of having a higher metabolic ‘ceiling’ (e.g. [21]). This relationship between metabolic rate and behaviour has been observed in species such as mosquito fish (*Gambusia affinis*) [22], deer mice (*Peromyscus maniculatus*) [23], Ouachita dusky salamanders (*Desmognathus brimleyorum*) [17] and zebra finches (*Taeniopygia guttata*) [24]. By contrast the *performance model* predicts that metabolic rate correlates with increased behaviours involved in energy gain. Under this model, individuals will have greater activity, be more dominant, and exhibit greater boldness if their metabolic rate is higher, owing to that individual's higher energetic requirements [13]. This interaction between behaviour and metabolism can be highly influential in the determination of dominance hierarchies, with certain personality types being more common in certain rank positions. In several species such as common lizards (*Zootoca vivpara)* [25], pied flycatchers (*Ficedula hypoleuca*) [26], great tits (*Parus major*) [26] and mountain chickadees (*Poecile gambeli*), for example, bold birds will occupy higher ranking positions, while opting to kleptoparasite subordinates instead of investing in foraging behaviours [27,28].

Cognitive abilities, broadly defined as the ability to acquire, process, store and use information from the environment [29], have not been studied in the context of metabolic rate but are linked to personality traits. For example, male guppies (*Poecilia reticulata*) that were bolder in the presence of a predator showed faster learning of an association between a visual cue and a food reward [30]. Boldness was also negatively correlated with decision-making time for sticklebacks (*Gasterosteus aculeatus*) in a T-maze [31]. Although investment in cognitive ability may enhance fitness-determining traits in some circumstances (e.g. [32–34]), investment into the formation and maintenance of neural networks in the brain probably comes at a constitutive cost to energy budgets [35,36], alongside potential induced energetic costs of use [37]. Metabolic rate may, therefore, play a role in determining energetic investment in memory traits.

Here, we explore the relationship between memory, metabolic rate, and personality. Memory is not a single trait, but comprises well-understood and physiologically distinct phases, one of which is short-term memory [38,39]. Short-term memory temporarily stores information for processing and decision-making and is an important component of cognitive ability, yet the relationship between an individual's metabolic rate and short-term memory capabilities is, as yet, unknown. We use juvenile female chickens (*Gallus gallus domesticus*) as a model system to directly link variation in aspects of cognition to metabolic rate, personality and dominance. In adult junglefowl, behavioural traits such as vigilance, habituation and speed of learning were found to play a role in social status and dominance, with variation in certain personality traits dictating the overall status of an individual within a group [40,41]. Specifically, we predict short-term memory to be ecologically relevant for chickens, as they must make decisions on patch quality when foraging, in which patch quality may dictate whether to continue to forage in that patch, or look for another patch resource [42]. These studies did not factor in metabolic demands, thus we aimed to take a holistic approach to understand how basal metabolic rate may determine cognitive performance (in this instance, short-term memory), and further possible interactions with personality and dominance.

We ask whether resting metabolic rate explains (i) short-term memory, (ii) two personality traits (boldness and neophobia), and (iii) dominance rank. Assuming that all these behaviours result in a net-positive energy gain for the individual, it is likely that metabolic rate will affect behaviour in one of three ways [13]: (i) individuals with a higher metabolic rate will show higher boldness and dominance behaviours and better short-term memory, because a higher metabolic rate will allow them to allocate a greater proportion of energy to these traits (performance model); (ii) there will be no relationship between metabolic rate and behaviours that result in a net-positive energy gain (allocation model); or (iii) behaviours that increase energy intake may be positively associated with metabolic rate (independent model). Additionally, given previous evidence of links between personality and cognitive traits in this and other species, we explore the relationship between personality traits and short-term memory in juvenile chickens, independently of metabolic rate.

## 2. Methods

### 2.1. Birds and husbandry

Twelve four-day-old female Goldline hybrid chickens were sourced from a private breeder (Cambridgeshire, UK). Chickens were chosen as the model species owing to the body of prior work that has categorized their behaviours, their well-understood and established husbandry requirements, and relative ease of access to their procurement. The birds were housed in a temperature-controlled room (Porkka, Royal Holloway University, Egham, UK) at 27°C, 45–50% relative humidity and a 12 L : 12 D cycle (11.00–23.00 light and 23.00–11.00 dark), for a period of six weeks. Chicks had access to a total floor space of 217 × 96 cm, including a brooder (approx. 35°C, Brinsea, UK) and perch space of 0.46 m chick^−1^. On arrival, chicks were fitted with different coloured elastic bands around the legs for ease of identification and at 16 days old had their wing feathers clipped (primary feathers 1–8) to prevent flying. Chicks had *ad libitum* access to water and chick crumbs (Baby Chick Crumbs, Small Holder Range, Allen & Page) when not undergoing metabolic rate measurements or behavioural observations, and their diet was supplemented with seeds and green vegetables. At six weeks of age, chicks were moved to an outdoor shed enclosure (360 × 240 cm) with a natural daylight pattern and ambient temperatures. Birds were weighed regularly to monitor their health. The experiment took place when chicks were 13 to 49 days old (electronic supplementary material, figure S1).

### 2.2. Measurement of resting metabolic rate

Resting metabolic rate (RMR) is the metabolic rate of an animal at rest, in a post-absorptive state and in its thermo-neutral zone [43]. Open-flow respirometry was used to measure RMR (rate of oxygen consumption ($\dot{V}O_{2}$) and carbon dioxide ($\dot{V}{\mathrm{CO}}_{2}$) production per minute). RMR measurements were taken from days 21 to 41 of the experiment, approximately every 2 to 3 days for each chick, such that the total number of metabolic traces per chick for the duration of the experiment was six or seven.

Chicks were initially habituated to the respirometry chambers over one week (four or five times per chick). On the evening before respirometry trials were conducted, food was removed (approx. 23.00–08.00, a minimum of nine hours), so that chicks were in a post-absorptive state. Access to water remained unrestricted. The following morning after food removal, three chicks were caught from their enclosure, transported to the laboratory, and placed into an individual respirometry chamber (31 × 23 × 23 cm, volume 12 l, opaque plastic), which contained two fittings through which incurrent air could be passed and excurrent air removed (electronic supplementary material, figure S2).

Prior to each session, the ambient O_2_ concentration was calibrated to 20.95% (based on ambient air; [44]). Each respirometry session was started by measuring an ambient air baseline as a reference (20 min), followed by each of the three chicks (20 min each), before ending with another ambient air baseline (10 min). Ambient air was pulled through the chamber at a flow rate of approximately 1600 ml min^–1^ (mean ± s.e.m. 1616.85 ± 4.80 ml min^–1^; SS-4 Sub-Sampler Pump, Sable Systems, Las Vegas, USA), making the flush-out rate of the chamber approximately 8 min. Chicks remained in their chambers for the duration of the session (a total of 90 min), allowing time for them to settle before measurements were taken. The order in which chicks were measured was randomized each day. Respirometry was conducted at 24.06 ± 0.003°C (mean ± s.e.m.; minimum = 21.42°C, maximum = 25.89°C across all traces).

On leaving the chamber, air was passed through a humidity sensor (RH-300, Sable Systems), scrubbed of water vapour (anhydrous indicating Drierite, W. A. Hammond Drierite Co. Ltd, Ohio, USA) before entering the CO_2_ analyser (CA-10a, Sable Systems) and further scrubbed of water vapour and CO_2_ (soda lime, Sigma Aldrich, Merck KGaA, Darmstadt, Germany) before entering an O_2_ and temperature analyser (FC-10a, Sable Systems). Analysers were connected to a laptop via a UI-2 interface and data were logged in Expedata software (Sable Systems; electronic supplementary material, figure S2).

All respirometry experiments were performed between 07.00 and 12.00 GMT, such that chicks participating in respirometry trials that day were removed from the main enclosure before lights came on or food was replaced. This ensured that the chicks were placed in the chambers still in their rest phase and calm, and in a post-absorptive state. Work was also carried out under red light and with minimal human presence, to reduce disturbance to the chicks. Chicks had their body mass (Adventurer Analytical, Ohaus, NJ, USA; ± 0.01 g) and tarsus length (measured from the elbow joint to the ankle joint; J-Bonest Vernier Callipers, UK; ± 0.01 mm) measured directly after they had undergone a respirometry session.

Metabolic rate was calculated using the equations:

$$\dot{V}O_{2}(\mathrm{ml}\;\min^{-1})=\;\frac{\mathrm{incurrent}[O_{2}]-\mathrm{excurrent}[O_{2}]}{1-\mathrm{incurrent}[O_{2}]}\;\times \mathrm{flow}\;\mathrm{rate},$$

for calculating the rate of O_2_ consumption, and:

$$\dot{V}CO_{2}(\mathrm{ml}\;\min^{-1})=\;\frac{\mathrm{excurrent}[{\mathrm{CO}}_{2}]-\mathrm{incurrent}\;[{\mathrm{CO}}_{2}]}{1-\mathrm{incurrent}[{\mathrm{CO}}_{2}]}\;\times \mathrm{flow}\;\mathrm{rate},$$

for calculating the rate of CO_2_ production, where incurrent gas is a measure of the ambient baseline, excurrent gas is a measure of gas on leaving the animal chamber, and flow rate denotes the rate at which ambient air was pulled through the system.

For each trace of RMR, $\dot{V}O_{2}$ and $\dot{V}{\mathrm{CO}}_{2}$ were averaged across a 5 min period between minutes 13 to 18 of the 20 min trace, which ensured the chick had settled in the chamber and air in the chamber had been fully replaced at least once. For any traces that appeared unstable during this period (O_2_ or CO_2_ peaks, or high oscillation), the nearest stable 5 min was selected. Traces were corrected for any shift in ambient baselines (difference in O_2_ and CO_2_ concentrations between the final and initial ambient baseline readings). All calculations were computed in Matlab v2019a [45].

### 2.3. Short-term memory assay

Short-term memory was assessed using an analogue of the radial arm maze (RAM), originally developed to test pharmacological effects in rodents [46] and since adapted for use in multiple species (e.g. [47]).

**Figure 1.**
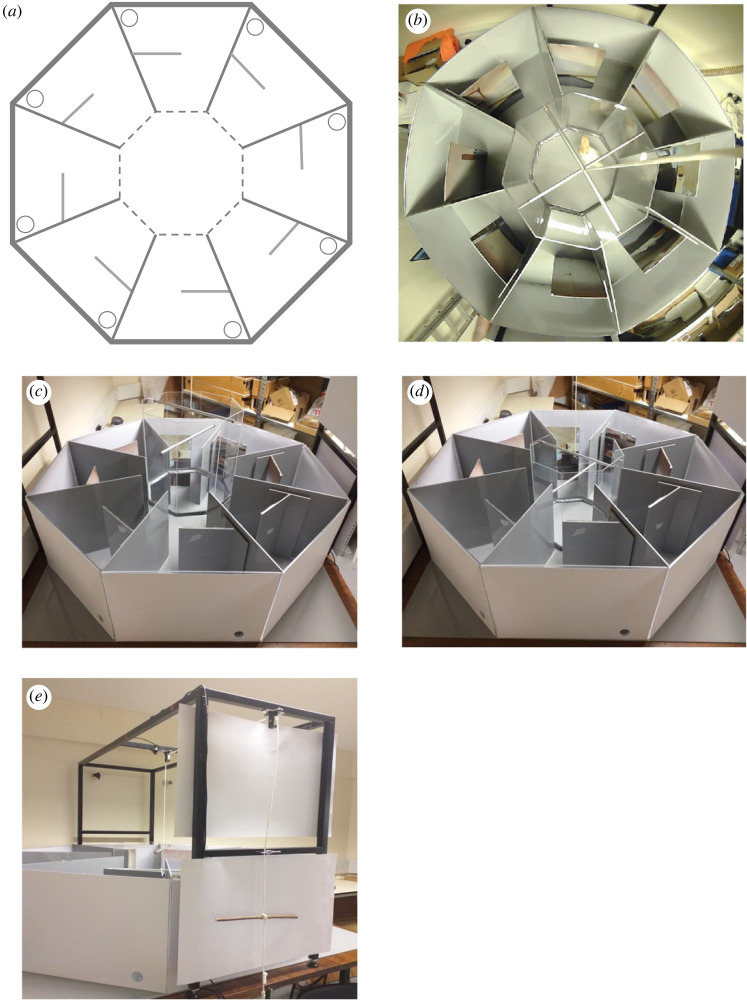
The radial arm maze (RAM) that was used to assay short-term memory in juvenile chickens. (*a*) Diagram of the aerial view of the RAM, including the additional wall half-way down each arm (solid grey line), food dish (grey circle), and central locking mechanism (smaller dashed octagon) that confined chicks to the centre of the maze for 10 s between each visit. (*b*) Aerial photograph of the maze with a chick confined to the centre; the maze walls appear curved as the photograph is taken using a wide-angle lens. (*c*) The central locking mechanism was operated using a pulley system attached to the outside of the RAM, and was opened to allow chicks to visit an arm and (*d*) closed to confine them to the centre. (*e*) The pulley system was attached to a frame around the outside of the RAM.

The RAM was octagonal, with a central platform (40 cm diameter) leading to eight arms (each 50 cm in length; figure 1*a,b*). A plastic divider was placed half-way down each arm on the left-hand side (40 cm × 20 cm), behind which there was a plastic food dish (6 cm diameter) hidden from view and baited with a single mealworm. A panoramic photo of the laboratory in which the maze was located was divided into eight sections and attached onto the dividers, providing the visual cues for chicks when inside the maze. The outside walls of the maze were solid white, so that the chicks were unable to see the experimenter and general surroundings. To discourage chicks from using stereotypic movements in the maze (i.e. movements of a fixed pattern, such as visiting arms consecutively clockwise), a central locking mechanism (CLM) was installed [48,49]. This consisted of a smaller clear octagon of the same diameter as the central platform attached to a pulley on a frame above the maze, which, when lowered, blocked the entrances to all eight arms (figure 1*c,d,e*). Each time a chick visited an arm and returned to the centre, the CLM was lowered, and the chick was held in the centre for 10 s. This (i) prevented immediate re-visits without the use of visual cues, and (ii) prevented stereotypic movement. After 10 s, the CLM was lifted 12 cm off the base of the maze, allowing the chick to visit another arm of its choice. Chicks were gradually acclimated to the experimental set-up and procedures (see the electronic supplementary material, Information).

Individual trials began when chicks were 26 days old, and continued for 14 consecutive days, such that each chick attempted a total of 14 trials. Food was withheld for a minimum of 30 min prior to a RAM trial, to increase motivation to complete the task. For each trial, a chick was caught from the enclosure**,** transported to the laboratory in a dark cardboard container and left to settle for a minimum of 5 min. The chick was then placed into the centre of the maze with the CLM fully closed. After 10 s, the CLM was lifted 12 cm from the base and the chick could enter any arm of its choice. The CLM was closed for 10 s each time a chick returned to the centre after visiting an arm. This continued until the chick had visited all eight arms or the trial had lasted for 30 min, whichever was reached first. Trials were stopped for any chicks showing signs of distress, or for chicks that attempted to fly out of the maze (36 out of 168 trials). A chick was considered to have visited an arm once its whole body had crossed under the CLM. Testing took place in the afternoons, and chicks were selected at random using a random number generator, to minimize the effect of time on testing. All trials were recorded using a 180° fisheye lens camera (ELP-USBFHD01M USB Camera) and Debut Video Capture Software (NHC, Canberra, Australia).

### 2.4. Personality assays

Personality traits (boldness and neophobia) were measured both pre- and post- exposure to the RAM (when chicks were 17–25 and 42–49 days old, respectively). Arenas were made of black and brown cardboard (pre-RAM, 90 × 70 cm), and an empty bird housing shed (post-RAM, 360 × 240 cm). All trials were filmed without the experimenter present in the room (EK7000 4 K Action Camera, Akaso, USA). Each assay (novel environment and neophobia) was repeated a total of six times (*n* = 3 pre-RAM and *n* = 3 post-RAM). Dominance was measured (see below) using the same filming equipment and arenas.

### 2.5. Novel environment exploration

Methods were based on assays of boldness in pigeons (*Columba livia;* [50,51]). For each trial, a chick was caught from its enclosure and transported to the arena. During pre-RAM trials, the chick was placed into a familiar shelter in the arena (19 × 23 × 15 cm) and left to settle for 2 min in the dark. The lights were then turned on and the chick was filmed for 10 min. During post-RAM trials, the lights could not be controlled, and so the chick was placed into a familiar shelter (48 × 30 × 30 cm), covered with a cloth and left to settle for 2 min. The cloth was then lifted, and the chick filmed for 10 min. To keep the arena novel between trials, the location of the shelter was moved to a different corner for each trial (electronic supplementary material, figure S3a). We used latency to leave the shelter as a measure of boldness [50].

### 2.6. Neophobia

Chicks were caught from their enclosure, placed into the arena and left to settle as in the novel environment trials. To control for confounding effects of a novel arena and novel object, we (i) performed all neophobia trials after novel environment trials had been completed, and (ii) allowed each chick an initial 5 min to familiarize themselves with the arena before each novel object trial began. Novel objects were placed on the furthest side of the arena opposite the shelter (electronic supplementary material, figure S3b; novel objects pre-RAM: Rubik's cube, baby rattle, coloured ball; post-RAM: coloured travel mug, purple dog toy, rubber hamburger toy). Chicks were filmed for 10 min with the novel object present in the arena, and we used latency to leave the shelter when the novel object was present as a measure of neophobia [50].

### 2.7. Dominance trials

Dominance was defined as the antagonistic interactions between conspecifics in which the winner is the dominant and the loser(s) are more subordinate [52]. All dominance trials took place in a group setting. Food was withheld for a minimum of three hours prior to trials starting. All 12 individual chicks were sequentially placed in an unfamiliar space. The space had the carrier placed in it at the start of the trial, and a food bowl placed on the opposing side of the carrier. The feeder was not covered and could allow for four to five chicks to comfortably feed in the same space. Following the opening of the carrier, the camera was started, and the experimenter exited the area.

The antagonistic interactions between individuals were recorded, covering the whole area, for 20 min. The interactions recorded were: shoving, pecking and wing flapping (following [50,53]). Shoving is defined as one individual using the whole body to push another, in which the receiver moves out of the way as a result. Pecking is defined as the use of the beak to bite another in an aggressive way. Wing flapping is the movement of wings when deterring another individual, undertaken in an aggressive manner. Additionally, time spent standing above or over the food bowl was recorded for each individual. Each chick was watched individually for the duration of the video and a tally was recorded for each interaction between conspecifics. The winner-loser matrix was then used to calculate a rank for each bird using David's score [54]. We performed six separate dominance trials in total (three trials between days 21–25 before chicks underwent short-term memory testing, and three trials after short-term memory testing, between days 47 and 49). All dominance trials were assessed by the same observer. All work was approved by the Royal Holloway Ethics Committee and did not require a Home Office Licence.

## 3. Data analysis

To correct metabolic rate for inter-individual differences in body size, each measure of O_2_ consumption or CO_2_ production was divided by the corresponding body mass of the chick taken directly after that metabolic rate measurement. Only body mass was used for these corrections, as body mass and tarsus length were strongly positively correlated (*r* = 0.98, *t* = 45.24, *p* < 0.001, *n* = 75; electronic supplementary material, figure S4). We first looked at whether raw and mass-corrected rates of O_2_ consumption ($\dot{V}O_{2}$; as a proxy for metabolic rate) change over time, using a linear mixed effects model (LMER) with the day of measurement as the predictor and chick as a random factor.

We used the number of errors made during a RAM trial as a measure of short-term memory capacity (fewer errors = predicted better short-term memory). Following Samuelson *et al*. [55], to determine whether chicks use their short-term memory in the RAM, we ran Monte Carlo simulations to calculate the predicted number of errors for a chick completing the RAM completely at random, with each arm carrying an equal probability of being chosen. Simulations were run with 10 000 iterations and compared with observed data using a one-sample *t*-test. We next looked at whether chicks use stereotypical movements (e.g. always move clockwise/anti-clockwise, always going to the opposite arm etc.) when completing the RAM. We created a matrix of probabilities of moving to an arm of the maze based on which arm had just been visited (probabilities calculated across all RAM trials and all chicks). We used this matrix in a second set of Monte Carlo simulations (10 000 iterations) to predict whether stereotypical movement rules explained RAM performance.

We created a generalized linear mixed effects model (GLMER) to look at whether the number of errors made during a RAM trial changed over time, with trial number as the predictor and chick as a random factor, and a negative binomial distribution (link function = ’logit’) to account for overdispersion.

The repeatability of personality traits was assessed by calculating the intraclass correlation coefficients, which is the proportion of variance explained by the random effect of individual identity in a model with no fixed predictors. The significance of repeatability was assessed using likelihood ratio tests and the 95% repeatability was estimated using 10 000 parametric bootstraps [56]. We included individual chick and block as random effects, to assess whether personality traits were repeatable for individual chicks between trials (chick), and whether they were repeatable before versus after chicks had undergone short-term memory testing in the RAM (block). We ran the model separately for boldness and neophobia traits. Chicks that did not leave the shelter during a trial were assigned the maximum time of 600 s.

To assess whether metabolic rate may explain short-term memory, personality traits or dominance, we used model Akaike information criterion (AIC) values to determine the model that best explained the variance in our data. For each analysis, we created a null model, and then added mean mass-corrected $\dot{V}O_{2}$ (metabolic rate) as a fixed factor, to determine whether it improved the model fit. Model fit was assumed to be improved if the ΔAIC was ≥2.00 [57,58]. In cases where the ΔAIC was less than 2.00 we selected the simpler (null) model. For models of short-term memory, we used a GLMER with errors made in a trial in the RAM as the response, chick as a random factor and a negative binomial error structure. For models of personality (boldness and neophobia), we used survival analysis models with latency to leave the shelter as the response, and a random error structure of by-individual random slope for metabolic rate and by-block random slope for metabolic rate. The survival model accounted for chicks that did not leave the shelter during a trial. To assess whether mass-corrected metabolic rate may explain dominance behaviours, we first created agonistic interaction matrices based on David's score [54], and subsequently used a linear model (LM) with David's score as the response variable.

Finally, we looked at whether personality traits may independently explain short-term memory performance or dominance score. For short-term memory performance, we used a GLMER with (i) scaled average latency to leave the shelter during exploration of a novel environment (boldness), and (ii) scaled average latency to leave the shelter during neophobia trials, as the predictors, RAM errors as the response, chick as a random factor and a negative binomial (link function = ‘logit’) error structure. For dominance, we used an LM with the same predictors as the model for short-term memory performance, and David's score as the response. We created a full model, null model, and a subset of models containing each variation of the fixed factors, retaining chick as a random factor in the mixed model. We then selected the model that best explains our data using AIC values.

Scaling was performed by centring the value on a mean of zero and standard deviation of one, using the following equation:

$$x^{\prime }=\frac{x-\mathrm{mean}\;\mathrm{of}\;\mathrm{group}}{\mathrm{standard}\;\;\mathrm{deviation}\;\mathrm{of}\;\mathrm{group}}\;,$$

where *x* was the initial measure of a variable and *x*’ was the scaled measure.

All analyses were conducted in R v. 4.2.1 [59] using the packages lme4 [60], lmerTest [61], car [62], survival [63], rptR [64], ggplot2 [65], RVAideMemoire [66] and coxme [67].

## 4. Results

### 4.1. Resting metabolic rate

**Figure 2.**
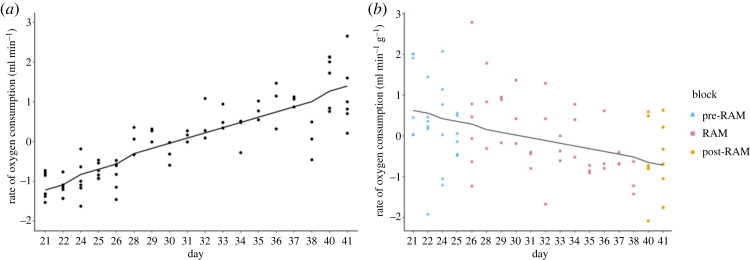
Resting metabolic rate across the experimental period (measured from days 21–41). (*a*) Scaled rate of oxygen consumption without mass-correction. Points show individual measurements; the line is drawn using points predicted from the model. (*b*) Scaled mass-corrected rate of oxygen consumption. Points show individual measurements in each of the three experimental blocks (blue triangle, pre-radial arm maze (RAM) measurements; pink square, measurements made on days when chicks underwent individual trials in the RAM; orange circle, post-RAM measurements); the line is drawn using points predicted from the model. Note days 23, 27 and 39 are missing as no metabolic rate measurements were taken on those days.

A total of 75 traces of RMR (rate of oxygen consumption, $\dot{V}O_{2}$ and rate of carbon dioxide production, $\dot{V}{\mathrm{CO}}_{2}$) were recorded, amounting to six or seven traces per bird between days 21 and 41 of the experiment (electronic supplementary material, figure S1). $\dot{V}O_{2}$ and $\dot{V}{\mathrm{CO}}_{2}$ were significantly positively correlated (*r* = 0.96, *p* < 0.001, *n* = 75; electronic supplementary material, figure S5), therefore only $\dot{V}O_{2}$ was used in subsequent analyses. $\dot{V}O_{2}$ significantly increased over time (LMER: time parameter estimate = 0.13, *t* = 19.03, *p* < 0.001; ΔAIC between full model and null model = 111.89; figure 2*a*). Mass-corrected $\dot{V}O_{2}$ was significantly different between chicks (ANOVA: *F*_11, 63_ = 2.28, *p* = 0.021) and significantly decreased over time (LMER: time parameter estimate = −0.07, *t* = −4.89, *p* < 0.001; ΔAIC between full model and null model = 11.64; figure 2*b*).

### 4.2. Does resting metabolic rate explain radial arm maze performance?

We used total number of errors made during a trial in the RAM as a measure of short-term memory. In total, 11 of the 12 chicks tested engaged with the RAM (one chick persistently attempted to fly out and did not complete any trials), completing 132 successful individual trials within the maze across the 14-day testing period. We first looked at how the performance of chicks in the RAM compared with performance simulated at random, or performance using stereotypical movement rules. Overall, chicks completed the maze better than would be predicted if choices were made randomly, making, on average, fewer numbers of errors in a trial than predicted at random (*t*-test, *t*_131_ = −3.05, *p* = 0.001; observed mean ± s.e.m. number of errors per chick = 11.64 ± 0.65, simulated mean ± s.e.m. number of errors per chick = 13.61 ± 0.09; electronic supplementary material, figure S6a). Similarly, chicks made fewer errors in the RAM than predicted by using purely stereotypical movement rules (*t*-test, *t*_131_ = −6.27, *p* < 0.001; simulated mean ± s.e.m. number of errors per chick = 15.68 ± 0.10; electronic supplementary material, figure S6b, S7). The number of observed errors made during a trial did not change over time (including time did not improve the model fit; ΔAIC = 1.80, therefore we accept the simpler null model).

**Figure 3.**
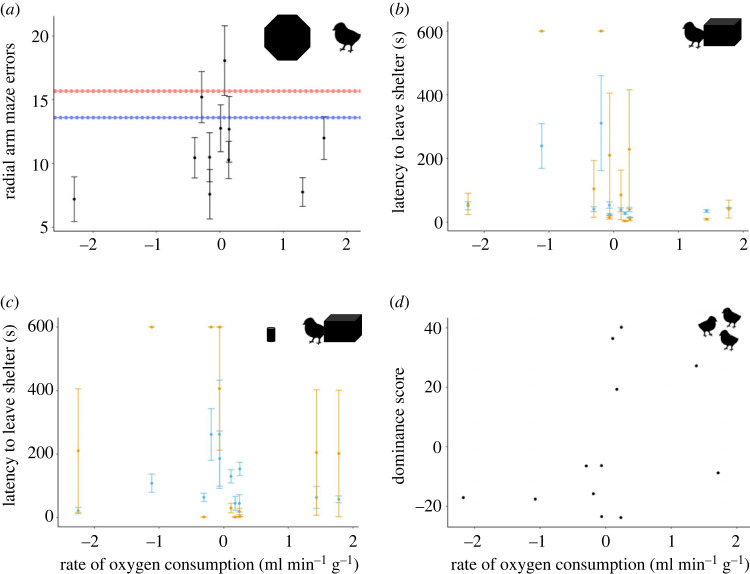
Relationship between scaled mean mass-corrected rate of oxygen consumption (RMR) and: (*a*) mean ± s.e.m. of total errors made in trials in the radial arm maze (RAM) trial, with lines showing mean (solid line) ± s.e.m., (dashed lines) simulated predictions if the RAM was solved by chance (blue), or using stereotypical movement rules (red); (*b*) mean ± s.e.m. latency to leave the shelter during boldness trials (blue points are trials completed pre-RAM exposure, yellow points are trials completed post-RAM exposure); (*c*) mean ± s.e.m. latency to leave the shelter in the presence of a novel object (neophobia; blue points are trials completed pre-RAM exposure, yellow points are trials completed post-RAM exposure); and (*d*) dominance (David's score) of each individual chick scored across six trials. Points show individual chicks (*n* = 11 in (*a*), *n* = 12 (*b–d*)). Chick icon made by Freepik from www.flaticon.com.

Mean mass-corrected $\dot{V}O_{2}$ did not explain the number of errors made by an individual during a RAM trial (including $\dot{V}O_{2}$ did not significantly improve the final model; ΔAIC = 1.38, therefore we accept the simpler null model; figure 3*a*). This did not change when chicks that performed worse than predicted by chance or stereotypical movement (*n* = 2) were removed (again, including $\dot{V}O_{2}$ did not significantly improve the final model; ΔAIC = 0.33, therefore we accept the simpler null model).

### 4.3. Does resting metabolic rate explain personality traits?

Both boldness and neophobia traits were repeatable across trials for each individual chick (boldness repeatability: *R* = 0.45 ± 0.14 (s.e.), 95% confidence interval (CI) = 0.12 to 0.66, *p* < 0.001; neophobia repeatability: *R* = 0.31 ± 0.13, 95% CI = 0.03 to 0.53, *p* < 0.001). However, personality trait measures appeared to differ when tested pre- or post- short-term memory testing in the RAM, with higher latencies to leave the shelter post-RAM testing driven by a higher number of individuals that did not leave the enclosure for the duration of a trial (boldness personality trials in which chicks did not leave their enclosure pre-RAM *n* = 1, post-RAM *n* = 7 out of *n* = 71 trials; neophobia personality trials in which chicks did not leave their enclosure pre-RAM *n* = 1, post-RAM *n* = 14 trials out of *n* = 72 trials). Latency to leave the enclosure in a novel environment was higher post-RAM testing (mean latency pre-RAM = 76.83 ± 114.71 s (s.d.), post-RAM = 151.28 ± 235.96 s) and was not repeatable between the two blocks (*R* = 0.01 ± 0.02 (s.e.m.), 95% CI = 0.00 to 0.06, *p* = 0.40). Similarly, latency to leave the enclosure in the presence of a novel object (neophobia) was higher post-RAM testing (mean latency pre-RAM = 116.44 ± 121.58 s, post-RAM = 239.91 ± 291.54 s) and was also not repeatable between the two blocks (*R* = 0.03 ± 0.03, 95% CI = 0.00 to 0.11, *p* = 0.20).

Boldness and neophobia traits were positively correlated (*r* = 0.40, *t* = 3.62, *p* < 0.001), but this appeared to be driven by chicks that did not leave their enclosures, with no correlation between the traits when these trials were removed from the analysis (*n* = 17 data points removed from the analysis, *r =* 0.20, *t* = 1.47, *p* = 0.15; electronic supplementary material, Results and figure S8). Boldness was not explained by mean mass-corrected $\dot{V}O_{2}$ (survival analysis, including mass-corrected $\dot{V}O_{2}$ did not significantly improve the final model, ΔAIC = 0.04; figure 3*b*). Neophobia was also not explained by mean mass-corrected $\dot{V}O_{2}$ (survival analysis, including mass-corrected $\dot{V}O_{2}$ did not significantly improve the final model, ΔAIC = 0.56; figure 3*c*).

In total, we recorded 933 interactions between the 12 chicks across six separate dominance trials. Dominance was not explained by metabolic rate (including metabolic rate did not significantly improve the final model, ΔAIC = 0.23; figure 3*d*). Separately, dominance was not explained by mean latency to leave the shelter during novel environment (boldness) and novel object (neophobia) trials (all models ΔAIC ≤ 1.36, accept the simplest (null) model). Personality traits (boldness or neophobia) did not explain the number of errors made in the RAM (all models ΔAIC ≤ 2.29, null model has the lowest AIC).

## 5. Discussion

Here, we tested whether RMR explained (i) short-term memory, (ii) two personality traits (boldness and neophobia), and (iii) dominance, in juvenile chickens. We found no relationship between metabolic rate and short-term memory, personality traits or dominance. Furthermore, there was no correlation between boldness and neophobia, and these traits did not predict short-term memory performance in the RAM.

### 5.1. Does resting metabolic rate explain short-term memory and personality traits?

Drawing conclusions about the energy-management strategy of chickens first requires discussion around what is being tested in the RAM. The RAM has previously been modified to measure foraging ability, particularly amongst nectivorous species [55,68] where individuals are expected not to re-visit a location as the resource is likely to have been depleted, thus leading them to adopt a win-shift foraging strategy. Assuming that chickens forage using a win-shift strategy, and are, therefore, performing a behaviour that should result in a net energetic gain to the individual, the lack of a relationship between metabolic rate and RAM performance can be explained by the allocation model. The allocation model predicts that metabolic rate does not determine the energy available for behaviour, so behaviours that result in energetic gain will not covary with metabolic rate [13,43]. However, adopting a win-shift strategy may not be ecologically relevant for chickens, which are likely to encounter dense clusters of food rather than resources that deplete in a single visit. Sulikowski & Burke [69] found miner birds (*Manorina melanocephala*) altered their foraging strategy depending on reward type in an open-field RAM. When arms were baited with nectar, the birds predictably used a win-shift strategy. However, a win-stay strategy was adopted when arms were baited with insect rewards [69], suggesting resource type may influence the way in which individuals approach the RAM task.

Differences in RAM performance have also been shown between species with different food storage strategies. For example, birds more reliant on seed-caching for survival (e.g. Clark's nutcrackers *Nicifraga columniana* and pinyon jays *Gymnorhinus cyanocephalus*) outperformed species less reliant on caching (scrub jays *Aphelocoma coerulescens* and Mexican jays *Aphelocoma ultramarina*) in a RAM analogue [70]. Such differences suggest ecological relevance of the RAM task is important to consider when deciding about behavioural strategies adopted by individuals. The RAM, therefore, may not be equivalent to a foraging task for chickens and so may not be associated with a behaviour that has net-energetic gain, but rather an energetically neutral behaviour, where there are both associated costs (i.e. performing the behaviour) and associated benefits (i.e. the potential to find a reward). The lack of a clear relationship between metabolic rate and energetically neutral behaviours (i.e. behaviours that do not result in an immediate net gain or net loss of energy) is predicted by the independent energy-management model, in which metabolic rate is independent of activity [71]. In keeping with this, we also found no relationship between boldness or neophobia and metabolic rate in the present study. These findings were consistent with a recent meta-analysis, which revealed that such energetically neutral behaviours, or behaviours which have no clear consequences for net energetic gain or loss, have no relationship with metabolic rate across 48 species [13].

Individuals with a relatively higher dominance rank are considered to be at an advantage, with relatively higher access to food resources compared with more subordinate individuals, resulting in a net-positive energetic outcome [13]. However, we found no relationship between dominance behaviours and metabolic rate, suggesting either that individuals with higher metabolic rates do not allocate more energy to dominance behaviours (allocation model), or that dominance in this scenario was not a behaviour with net energetic gain, but rather an energetically neutral behaviour (independent model). This is likely, given that chicks had access to ad libitum food at all times when not undergoing behavioural or respirometry trials.

### 5.2. Other variables influencing radial arm maze performance

One factor that may have affected performance during RAM trials could be differing motivation between individuals; motivation has previously been suggested as a non-task related driver of task completion in studies of cognition [72–74]. A common way to standardize motivation is to maintain individuals at a pre-determined percentage of their original body mass, or to withhold food for a standardized period of time prior to the task [73]. The former was not possible during the present study, as the chicks were less than six weeks old, so restricting food intake over a prolonged time period would have restricted growth rate, with potential implications on both metabolic rate and behaviour. Food was, therefore, withheld for all chicks for a minimum of 30 min prior to RAM trials. Despite this method being common practice, the effects of withholding food will depend on metabolic rate and digestion speed, as individuals with a relatively higher metabolic rate at rest require higher intakes of food to maintain their higher energetic costs [75]. Withholding food may, therefore, not be a suitable method for standardizing motivation. An alternative way to control for motivation could be to conduct motivation tests before a task and control for it during analysis, for example by measuring latency to approach a free food source (e.g. [74,76]) or through latency to approach the task apparatus out of choice (e.g. [77]). Using these latencies as proxies for motivation should be considered for future studies.

While the RAM has been considered as a test of short-term, or working, memory, the task can also be solved using stereotypical movement rules or random chance [55]. In our experiment, nine out of 11 chicks made, on average, fewer errors than predicted by chance or using stereotypical movement rules, suggesting that a degree of short-term memory was used to solve the task. However, when looking at individual simulation outputs (electronic supplementary material, figure S6), the observed mean number of errors made across all trials falls in the centre of the simulation curve, so we cannot be certain that chicks exclusively used their short-term memory to solve the maze. Consequently, we suggest that the RAM may not be the most suitable task on which to assay short-term memory in juvenile chickens. The exact duration of information storage in short-term memory is unknown; estimates range from a few seconds to several minutes [78], and up to four hours in rats (*Rattus* spp.; [79]), and appear to be individual- and context-dependent. One way in which this could be explored further is by increasing the confinement period (minutes to hours) of an individual in the centre of the maze at a half-way point during a trial, thus pausing and then restarting the task [49]. This would provide evidence for the length of time short-term memory is able to persist.

### 5.3. Personality and short-term memory ability

Personality has been suggested as a driver of differences in cognitive abilities between individuals [80,81]. Bolder individuals that show higher levels of boldness may encounter more learning opportunities, or may show higher activity levels and, therefore, approach, survey and solve a task faster [7,9,82]. Bolder individuals could, therefore, be predicted to make fewer errors in the RAM, as they may be more likely to enter novel (previously unvisited) arms. Conversely, we found no relationship between the level of an individual's boldness or neophobia, and errors made in the RAM. Several other studies have also found no evidence for a relationship between personality and cognitive traits. For example, Cole *et al*. [83] found boldness and neophobia did not explain problem-solving ability (manipulating a lever to gain a food reward) in great tits. Instead, some variation was explained by age and place of birth, with younger and non-native individuals more likely to solve the task [83].

One criticism of studies that test relationships between cognitive task performance and personality has been that they confine variables to a two-dimensional plane, where there are likely to be other unknown or unaccounted for variables acting on them [81]. Learning and memory, as well as personality traits, may be driven in part by the genotype [84], or by previous experiences of individuals, which are often difficult to control for when testing adult populations. Here, we controlled for age, sex, handling, previous experiences, and parental effects, as chicks were raised from hatching in the laboratory, strongly suggesting a genetic driver for differences between individuals. Future work could look to incorporate family lineages into analyses, as well as genotyping individuals, which would show whether variation in behaviour correlates with variation in the genotype.

In summary, we found that metabolic rate at rest did not explain short-term memory performance, personality traits or dominance ranks in juvenile female domestic chickens. Our results provide support for the independent energy-management hypothesis, suggesting that energy is allocated to behaviours independently of an individual's metabolic rate. Previous work has suggested that behaviours, such as exploration of novel environments and novel objects, which are not associated with an overall net energetic loss or gain, are likely to show no relationship with metabolic rate [13], and are consistent with our results. The lack of a relationship between metabolic rate and short-term memory may suggest that the RAM does not represent a foraging task for chicks (i.e. a task with a net energetic gain), but is perhaps a test of boldness.

Future studies working to assess relationships between metabolic rate and behaviour could look to include multiple behavioural assays that test behaviours with known energetic profiles (i.e. a behaviour with net energetic loss, net energetic gain, and an energetically neutral behaviour), to further assess whether the relationship between metabolic rate and behaviour is dependent on the ecological function and energetic profiles of the behaviours.

### 5.4. Links to previous research and future directions

In the present study we found no association between metabolic rate and (i) performance in a short-term memory task, or (ii) personality traits and dominance. Our relatively small sample size of 12 birds precluded us from investigating all interactions within the models, instead focusing on main effects. It is possible, therefore, that the relationships between metabolic rate and the traits we measured are more intricate than our model permitted us to investigate. Thus, further studies with sufficient sample sizes to investigate all possible interactions would be beneficial.

Previous studies on red junglefowl have often focused on the behaviours of males rather than females, making direct comparisons between our study and previous studies potentially difficult. While Sorato *et al*. [85] demonstrated that, overall, the behavioural and cognitive capabilities of young male and female junglefowl were not statistically different, Zidar *et al*., [86] found that female chicks learnt faster than male chicks in a learning reversal task. Moreover, Zidar *et al*. [86] found that adult females differed in their exploration responses compared to males and juvenile females, suggesting a fruitful further study would be to determine the relationship between RAM performance and RMR in female chickens from juvenile into adulthood, to determine if these relationships alter as the birds mature. Another key difference found between male and female adult junglefowl was that females were more behaviourally flexible than males, with males being more persistent. Repeating our study with male chicks would make a worthwhile comparison to our findings in the present study with juvenile females, to determine at what point possible differences between males and females become evident.

Favati *et al*. [40] ascertained a link between the degree of vigilance and the likelihood of obtaining a dominant social position in junglefowl. A fruitful further investigation would be to determine if individual cognitive capabilities determined via a RAM are also linked to greater vigilance and potentially a greater RMR, and ultimately a higher social position and more offspring. Previous findings have shown that in junglefowl, there is no concept of a ‘smarter’ individual, but rather different individuals performed better than others on specific tasks [86]; faster-learning birds in one task, for example, did not learn quicker in another. Zidar *et al*. [86] suggested that the relationship between cognition and personality is, therefore, context-dependent and will differ depending on the task. Future work should expand the link between RMR, personality and dominance to a wider range of cognitive tasks in addition to a RAM. Such tasks previously studied in both chickens and junglefowl include reversal learning, discriminative learning and habituation [87,88]. In addition, many prior studies relate to learning speed, rather than accuracy, which contrasts with the RAM where the number of errors is the focus, rather than the speed of decision-making. Future work could include measuring time to completion of the RAM task, and thus factor in a potential speed-accuracy trade-off and its possible link to RMR.

## Data Availability

All data are available as an electronic supplementary material file [89]. All data and code are available at: https://figshare.com/s/be8259b99ca4faa88c70 [90].
